# A Rare Triad of Congenital Facial, Abducens, and Hypoglossal Nerve Palsies: A Case Report

**DOI:** 10.7759/cureus.98120

**Published:** 2025-11-29

**Authors:** Soumi Kundu, Winnie Koshy Binoj, Sarthak Das, Pradosh Kumar Sarangi, Saroj Kumar Tripathy, Md. Ehtesham Ansari, Archana Malik

**Affiliations:** 1 Pediatrics, All India Institute of Medical Sciences, Deoghar, Rampur, IND; 2 Radiodiagnosis, All India Institute of Medical Sciences, Deoghar, Rampur, IND; 3 Pulmonary Medicine, All India Institute of Medical Sciences, Deoghar, Rampur, IND

**Keywords:** abducens nerve, cranial nerve palsy, facial nerve, hypoglossal nerve, moebius syndrome, mri

## Abstract

Moebius syndrome is a rare congenital neurological disorder characterized primarily by nonprogressive facial and abducens nerve palsy. Involvement of additional cranial nerves is uncommon. We describe an eight-year-old boy presenting with left-sided facial nerve palsy, hypoglossal nerve atrophy, and absence of the abducens nerve. Clinical examination revealed facial deviation, restricted ocular abduction, tongue atrophy, and feeding and speech difficulties - features consistent with a mild Moebius spectrum. MRI demonstrated hypoplasia of the left facial nerve, absence of the left abducens nerve, and atrophy of the right half of the tongue, confirming hypoglossal involvement. This case underscores the importance of early recognition and detailed MRI evaluation to detect atypical cranial nerve anomalies in children with Moebius syndrome.

## Introduction

Moebius syndrome, initially described by Von Graefe and later defined by Paul Julius Moebius [1], is an uncommon congenital disorder predominantly involving the facial (VII) and abducens (VI) nerves, which control facial expression and ocular abduction, respectively [2]. The diagnosis is based primarily on clinical assessment, although recent studies have identified genetic associations [3].

Due to the absence of standardized diagnostic criteria, clinical evaluation and prognosis can be challenging. In 2007, experts at the Moebius Syndrome Foundation meeting in Bethesda, USA, defined the condition as “congenital, uni- or bilateral, nonprogressive facial weakness with limited abduction of one or both eyes” [4]. The condition may be unilateral or bilateral and can vary in severity. Other cranial nerves - including the hypoglossal (XII), glossopharyngeal (IX), vagus (X), and trigeminal (V) - may also be affected, producing difficulties in feeding, speech, and swallowing [5].

The etiology of Moebius syndrome is heterogeneous, involving both genetic and environmental factors. The vascular disruption hypothesis suggests impaired blood flow during embryogenesis (four to seven weeks of gestation) as a key mechanism. Teratogenic exposure to misoprostol, benzodiazepines, alcohol, or cocaine has also been implicated [6]. Mutations in the PLXND1 and REV3L genes, as well as chromosomal rearrangements between chromosomes 1 and 13, have been identified as potential genetic causes [7].

We present a case of an eight-year-old boy with congenital unilateral facial and abducens nerve palsy, accompanied by hypoglossal nerve involvement, highlighting the extended clinical and radiologic spectrum of Moebius syndrome.

## Case presentation

An eight-year-old boy, the third child of non-consanguineous parents, presented with facial asymmetry and unclear speech. Pregnancy and delivery were unremarkable, with no reported maternal illness, teratogenic exposure, or perinatal complications. Facial deviation and incomplete closure of the left eye were noticed shortly after birth and persisted during sleep. Feeding was difficult due to poor sucking, and the left eye showed reduced tearing during crying. Introduction of solid food was delayed, and speech development was slow. Developmental milestones and family history were otherwise normal.

**Figure 1:**
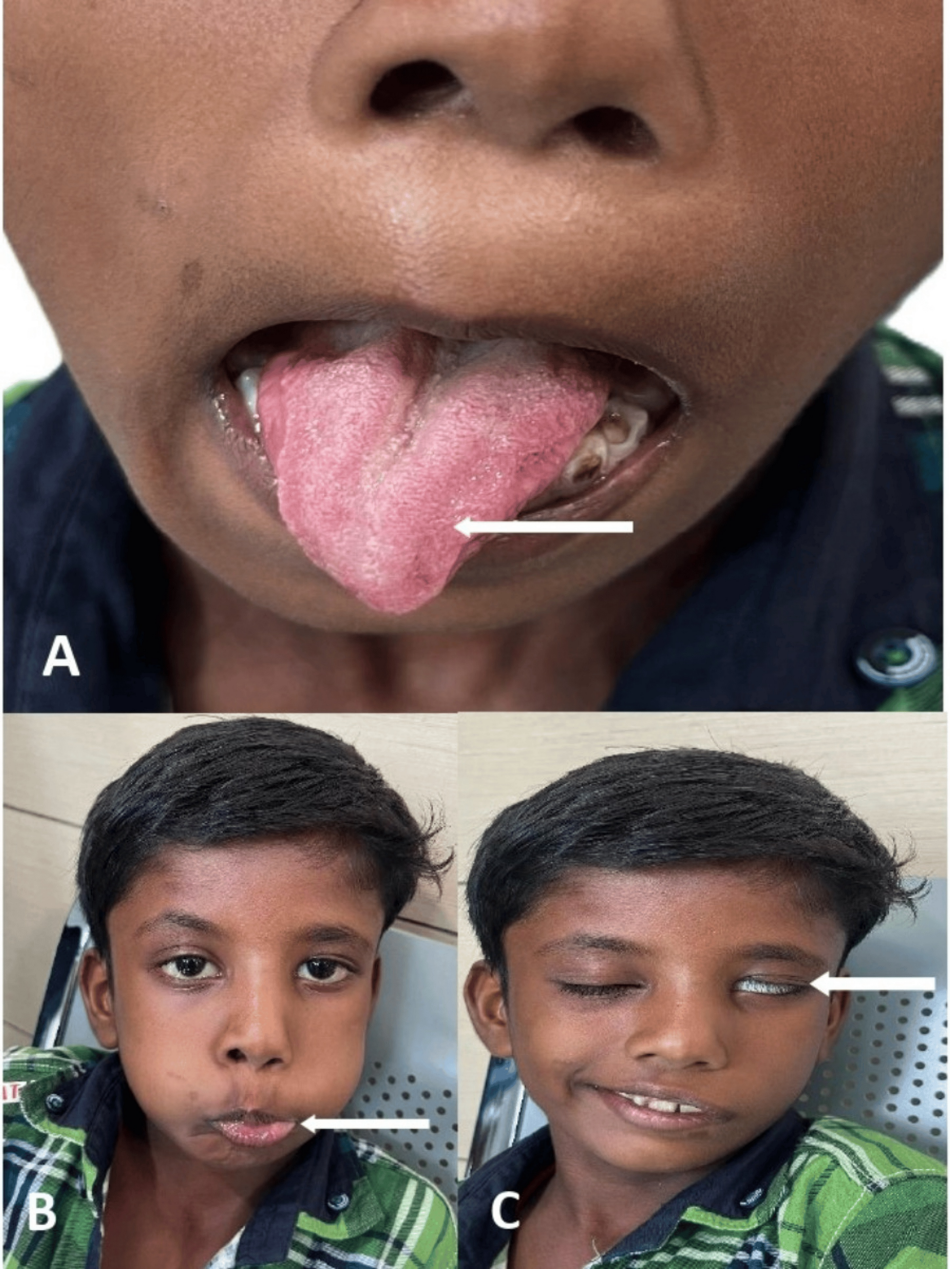
(A) Clinical photograph showing right-sided tongue atrophy with deviation of the tongue and angle of the mouth. (B) Difficulty in holding air within the mouth, and (C) inability to tightly close the left eyelid, indicate left facial nerve and right hypoglossal nerve palsy. The patient provided written and signed consent, allowing publication of this identifiable facial image in an open-access journal.

On examination, the child was alert and well-nourished. Facial asymmetry was noted, with deviation of the mouth to the right side. There was incomplete eye closure on the left, loss of the nasolabial fold, and inability to puff the left cheek - suggesting left facial nerve palsy. The left eye failed to abduct, indicating abducens nerve involvement. The tongue deviated to the right, with hemiatrophy of the right half, consistent with hypoglossal nerve palsy. Mild uvular deviation to the right suggested minimal vagus nerve weakness. Motor, sensory, and reflex examination findings were otherwise normal (Figure 1).

**Figure 2:**
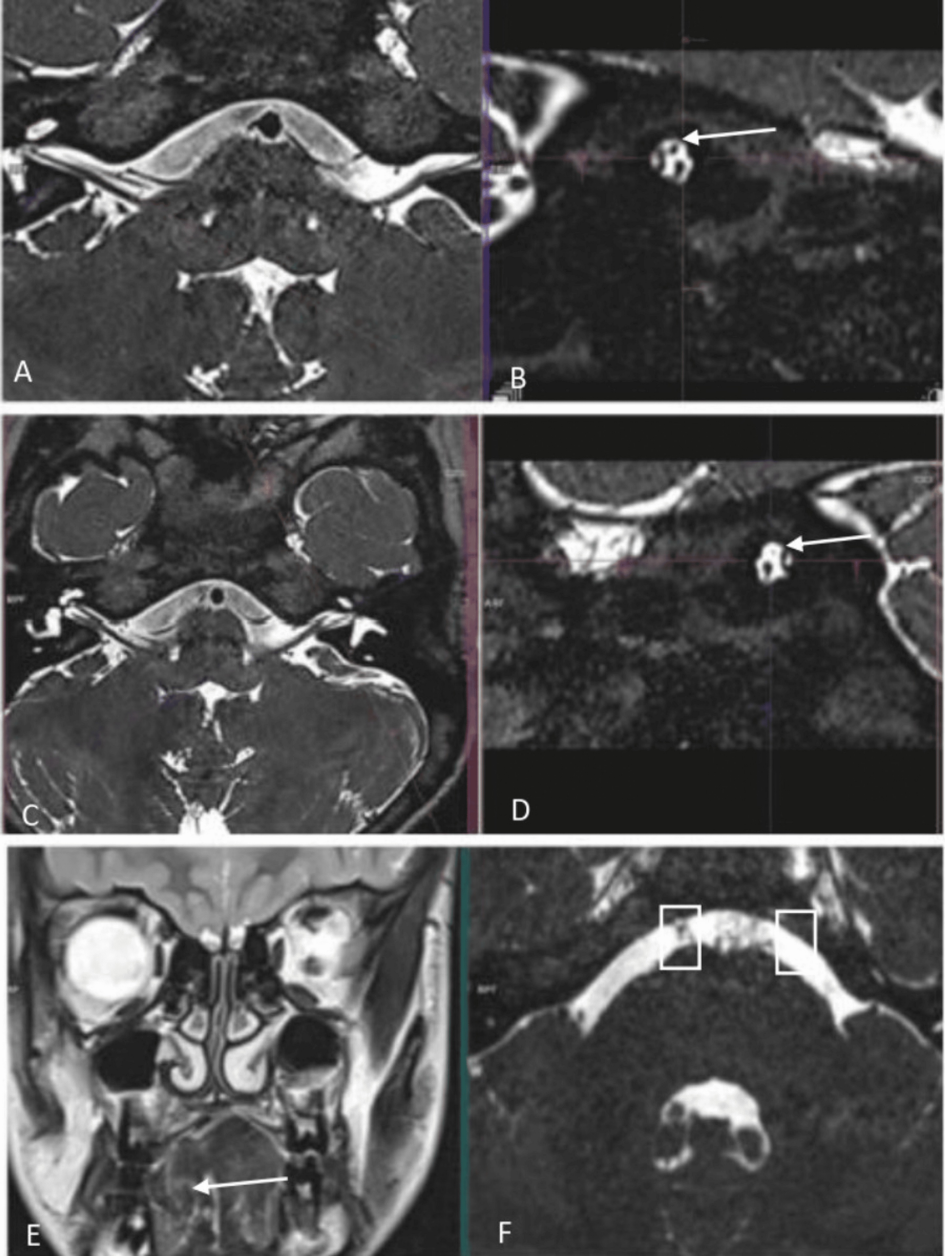
Axial (A) and oblique sagittal (B) 3D SPACE T2-weighted MRI images show the normal right facial nerve, located in the anterior superior quadrant of the internal auditory canal. (C, D) Marked hypoplasia of the left facial nerve is seen (arrow), while the right facial nerve appears normal. (E) Volume loss in the right half of the tongue (arrow), likely secondary to right hypoglossal nerve palsy. (F) A linear structure consistent with the right abducens nerve is visible (rectangle), while the left abducens nerve is not appreciable, suggesting congenital absence. CSF flow artifact is noted in the prepontine cistern. SPACE: Sampling Perfection with Application-optimized Contrasts using different flip-angle Evolutions

MRI of the brain revealed hypoplasia of the left facial nerve within the internal acoustic meatus, absence of the left abducens nerve, and right-sided tongue atrophy (Figure 2). These findings confirmed multiple cranial nerve deficits, consistent with Moebius syndrome.

The child was placed on a multidisciplinary rehabilitation plan, including physiotherapy and speech therapy. Eye protection measures, such as lubrication and nighttime eye closure, were advised. Over follow-up, gradual improvement in articulation and oral motor function was observed.

## Discussion

Moebius syndrome is defined by congenital, nonprogressive paralysis of the facial and abducens nerves [1,2]. Although bilateral presentation is more common, unilateral forms, as in this case, have been described [3]. Additional cranial nerve involvement broadens the clinical spectrum. Our patient exhibited facial, abducens, and hypoglossal nerve palsies, with mild vagus involvement, leading to tongue atrophy, speech impairment, and swallowing difficulties.

Facial nerve involvement occurs in almost all affected individuals, while abducens palsy is reported in about 75% of cases [4]. Hypoglossal nerve abnormalities are less frequent, observed in roughly 25% of patients [5]. MRI plays a critical role in visualizing absent or hypoplastic cranial nerves, supporting clinical diagnosis and aiding prognosis. High-resolution 3D constructive interference in steady state (CISS) MRI sequences are particularly effective in detecting the absence of facial nerves, allowing early confirmation of Moebius syndrome without unnecessary additional tests [8,9].

The underlying pathogenesis remains uncertain. Both vascular disruption during embryonic development and genetic factors affecting hindbrain formation are leading hypotheses [6,7]. No identifiable environmental or genetic cause was evident in our case.

Management is largely supportive and multidisciplinary. Early physiotherapy and speech therapy are essential to improve oral motor control and social communication skills. Surgical facial reanimation may be considered in selected cases. Ophthalmologic care remains vital to prevent exposure keratitis resulting from incomplete eyelid closure [10].

## Conclusions

This case highlights a rare presentation of Moebius syndrome with concurrent facial, abducens, and hypoglossal nerve palsies. MRI serves as a key tool in confirming cranial nerve deficits beyond clinical observation. Recognizing atypical presentations early and initiating comprehensive, multidisciplinary care can significantly enhance functional and developmental outcomes in affected children.

## References

[1] Monawwer SA, Ali S, Naeem R (2023). Moebius syndrome: an updated review of literature. Child Neurol Open.

[2] Broussard AB, Borazjani JG (2008). The faces of Moebius syndrome: recognition and anticipatory guidance. MCN Am J Matern Child Nurs.

[3] Bell C, Nevitt S, McKay VH, Fattah AY (2019). Will the real Moebius syndrome please stand up? A systematic review of the literature and statistical cluster analysis of clinical features. Am J Med Genet A.

[4] Miller G (2007). Neurological disorders. The mystery of the missing smile. Science.

[5] Picciolini O, Porro M, Cattaneo E, Castelletti S, Masera G, Mosca F, Bedeschi MF (2016). Moebius syndrome: clinical features, diagnosis, management and early intervention. Ital J Pediatr.

[6] Zaidi SM, Syed IN, Tahir U, Noor T, Choudhry MS (2023). Moebius syndrome: what we know so far. Cureus.

[7] Tomas-Roca L, Tsaalbi-Shtylik A, Jansen JG (2015). De novo mutations in PLXND1 and REV3L cause Möbius syndrome. Nat Commun.

[8] Verzijl HT, Valk J, de Vries R, Padberg GW (2005). Radiologic evidence for absence of the facial nerve in Möbius syndrome. Neurology.

[9] Herrera DA, Ruge NO, Florez MM, Vargas SA, Ochoa-Escudero M, Castillo M (2019). Neuroimaging findings in Moebius sequence. AJNR Am J Neuroradiol.

[10] Odedra A, Blumenow W, Dainty J (2024). Multidisciplinary care for Moebius syndrome and related disorders: building a management protocol. J Clin Med.

